# MAP, Johne’s disease and the microbiome; current knowledge and future considerations

**DOI:** 10.1186/s42523-021-00089-1

**Published:** 2021-05-07

**Authors:** Chloe Matthews, Paul D. Cotter, Jim O’ Mahony

**Affiliations:** 1 Cork Institute of Technology, Bishopstown, Co. Cork, Ireland; 2 Teagasc, Food Research Centre, Food Biosciences Department, Fermoy, Co. Cork, Ireland; 3 APC Microbiome Institute, University College Cork, Co. Cork, Ireland

## Abstract

*Mycobacterium avium subsp. paratuberculosis* is the causative agent of Johne’s disease in ruminants. As an infectious disease that causes reduced milk yields, effects fertility and, eventually, the loss of the animal, it is a huge financial burden for associated industries. Efforts to control MAP infection and Johne’s disease are complicated due to difficulties of diagnosis in the early stages of infection and challenges relating to the specificity and sensitivity of current testing methods. The methods that are available contribute to widely used test and cull strategies, vaccination programmes also in place in some countries. Next generation sequencing technologies have opened up new avenues for the discovery of novel biomarkers for disease prediction within MAP genomes and within ruminant microbiomes. Controlling Johne’s disease in herds can lead to improved animal health and welfare, in turn leading to increased productivity. With current climate change bills, such as the European Green Deal, targeting livestock production systems for more sustainable practices, managing animal health is now more important than ever before. This review provides an overview of the current knowledge on genomics and detection of MAP as it pertains to Johne’s disease.

## Introduction

### *Mycobacterium avium subsp. paratuberculosis* and Johne’s disease

Mycobacteria, which belong to the phylum Actinobacteria, are Gram-positive, acid-fast organisms that include a number of relevant human and animal pathogens such as *Mycobacterium leprae*, *Mycobacterium tuberculosis* and *Mycobacterium avium subsp. paratuberculosis* (MAP). MAP, the focus of this review, is a slow-growing, rod-shaped, non-spore-former, with a unique complex lipid cell wall structure [1]. It is this complex cell wall structure that is in part responsible for its persistence in the environment and in the host. MAP is an intracellular parasite of animals [2] and, paradoxically, as the growth rate of the microorganism decreases, its pathogenicity increases [1]. Unlike some other mycobacteria, MAP is unable to synthesize mycobactin, a siderophore employed to obtain iron from environmental sources. As a result, MAP cannot replicate outside of the animal host without mycobactin supplementation. This *Mycobacterium* is the causative agent of Johne’s disease (JD) in ruminants, a chronic enteritis, causing weight-loss and diarrhoea, increased susceptibility to other diseases, and economically impacts farmers worldwide [3].

MAP is most commonly transmitted during neo-natal life, after transmission via the oral route. Infected animals can be classified into a number of distinct stages of silent, subclinical (greatest threat to eradication and control), clinical and advanced stages of disease. These categories are based on the presence and severity of clinical signs, the rate of environmental shedding and the likelihood of detection with current MAP diagnostic methods [4]. Animals that are infected with MAP, but have no evidence of disease and cannot be detected with current diagnostic methods, are said to be in the silent stage [5]. Subclinical animals show an immune response, shedding low but infectious levels of the pathogen. Clinical and advanced stages are represented by diarrhoea and weight-loss to eventual death due to dehydration and cachexia [6]. It is important to distinguish between infection and disease as infection does not always equate to disease. The disease results in a decrease in milk yield (reported in both clinical and sub-clinical stages of infection), adverse effects on reproductive performance and increased culling rates.

The “iceberg phenomenon” allows the generation of estimates for JD prevalence. It is driven by the theory that there is an underestimation of prevalence and that the true prevalence in infected herds is greater than the apparent prevalence [7]. This was first described by Whitlock and Buergelt [4], (1996) where they reported that for every animal in the advanced stage of disease, it is assumed that there are one to two in the clinical stage, four to eight in the subclinical stage, and ten to fourteen in the silent stage. More recent advances in mathematical modelling have indicated that the number of subclinical shedding animals is higher than animals in the silent stage [7]. It is important to note that only 10–15% of animals that are infected progress to clinical disease, suggesting that most calves successfully clear the infection or they are resistant to the disease [8].

After oral ingestion of the pathogen, the tissue of the intestinal mucosa and submucosa of the ileum and jejunum, or more specifically the M cells of the Peyer’s patch, is targeted by MAP, allowing the subsequent invasion of, and multiplication within, intestinal macrophages. Gross pathological changes in cattle with JD include thickening and corrugation of the intestinal wall (resulting in an inability to absorb nutrients), dilation of lymphatic vessels and enlargement of mesenteric lymph nodes [9]. The bacilli can remain viable for extended periods of time in water, soil and faeces [10, 11], but can survive in urine and silage for much shorter periods due to the challenges of enduring high ammonia levels and low pH environments [11, 12].

Strains of MAP are notoriously difficult to isolate, often taking months to grow in pure culture, thereby making the detection and diagnosis of JD challenging. Upon histopathological examination, cases display granulomatous inflammation, primarily in the ileum and draining lymph nodes. Other sites along the gastrointestinal tract may also be involved. Clinical signs of the disease are not observed until around the third lactation [13, 14] or between 2 and 5 years after initial infection and can be characterised by loss of body condition, decrease in milk production and scour.

The importance of MAP with respect to the livestock sector in terms of economic losses (including reduced milk production, increased somatic cell counts, increased incidence of clinical mastitis, reduced fertility and increased susceptibility to other diseases) and welfare is further amplified by the potential zoonotic transmission of the bacteria [15, 16]. The economic impact of paratuberculosis in a cattle herd will depend on the number of animals affected, infected and infectious [16]. Animal health has also become a talking point in climate change mitigation strategies such as the European Green Deal as life cycle analysis suggests that greenhouse gas emissions in dairy cows with JD is up to 25% higher than healthy animals [17]. MAP has also been associated with a number of autoimmune diseases [1] in humans, including multiple sclerosis [18], type 1 diabetes [19] and Blau syndrome [20], with Crohn’s disease (CD) receiving the most attention due to the similarity of the symptomology of CD to JD and debate in relation to the potential for zoonotic transmission. MAP was first implicated in CD in 1913. CD is a complex immune-mediated chronic enteritis characterized by chronic gastrointestinal inflammation [21], with the gut microbiome being thought to be highly involved in pathogenesis with a reduced diversity and imbalance considered characteristic of the disease [22–24]. However, it is still unclear if inflammation is caused by specific taxa and the causative mechanisms have yet to be defined. In recent years, MAP has again been suggested as a possible cause of the disease as some studies have found that MAP strains can be cultured from the peripheral mononuclear cells from 50 to 100% of patients with CD [25, 26]. However, notably, MAP has also been found in healthy individuals with no clinical signs of infection [27, 28] and, although the zoonotic potential cannot be ignored, there is not yet enough evidence to either support or oppose an impact of MAP on public health [29], as Koch’s postulates have been fulfilled with respect to the involvement of MAP in ruminant, but not for human, disease.

Despite the lack of conclusive evidence to date, the potential for zoonosis is a concern. It is thus notable that MAP has been detected in milk from domestic animals using a number of detection methods [30–32]. MAP has been reported in raw milk in developed countries including, Czech Republic (2%), Ireland (0.3%), UK (6.9%), USA (0–28.6%) [33]. There is debate as to how well pasteurisation inactivates MAP [34–36], but it is important to recognise that the pasteurisation conditions used across studies vary considerably.

In this regard, rapid detection of mycobacteria from different matrices is necessary in order to prevent disease spread. Optimising tests, including standardisation and a capacity to work with small amounts of biological material where necessary, are all important developments that can be further improved. Utilising the microbiome in order to determine microbial biomarkers of exposure or infection may be pivotal for the control and prevention of JD going forward. As intestinal inflammation is one of the major traits of MAP infection and the resulting JD [3], having a major impact on the composition and functionality of the gut microbiome, it affects the host enormously in terms of nutrient absorption. The intestinal wall is thickened and the mucosal surface has thick, closely packed transverse folds [37]. The mucosa between folds may be reddened by congestion and ulceration. Lesions are seen at the site of infection in the gut, towards the terminal end of the ileum.

### Prevalence and distribution

Since the disease was first identified in the Netherlands, JD has been detected in all countries with ruminant populations, in both wild and domestic animals. The prevalence and distribution of JD varies from country to country. Estimates of true herd prevalence in Europe vary from 31 to 71% in The Netherlands [38], 47% in Denmark [39] and 18% in Belgium [40]. In Ireland, for example, prevalence of JD remains relatively low in comparison to other countries but has risen [41] as a consequence of Ireland joining the Single European market in 1992. This allowed the free movement of animals between European countries and resulted in the abandonment of pre-import testing and post-import quarantine.

Environmental sampling is a quick way to determine the presence of MAP in a herd without having to sample individual animals. However, this method is not sensitive enough for animals kept on open pastures and may only be used to detect the agent in housed dairy systems [42]. In a survey carried out by Good et al. (2009), it was found that, in Ireland, of over 20,000 animal samples tested, just 201 were classified as ELISA positive. 21.4% of herds had one or more ELISA positive animal, while only 6.4% had more than one ELISA positive animal [41]. The true prevalence of all animals tested in this study was found to be 2.86%.

### Management practices and infection control

MAP is present in the environment and can be found ubiquitously on farms with MAP positive herds. In a comprehensive survey on management practices by [43], it was found that many practices employed on Irish dairy farms impact on the transmission of JD. Although it has been suggested that a pasture-based system may lessen the prevalence of JD due to less exposure to contaminated faeces, practices such as importing animals and manure from other farms, pooling colostrum, using calving areas for more than one calving and housing sick animals in the calving area all contributed to higher increase in JD transmission. In a study describing calf to calf transmission, it was found that infection occurred due to exposure of infectious pen-mates to contact calves [44]. The study found that all animals were faecal culture positive (however, shedding stopped after animals were housed individually), 50% of contact exposed animals had MAP positive tissue results and 36% had evidence of a cellular immune response. Although the sample size of the study was small (*n* = 32), it did involve an intensive sampling regimen with 3 sampling time points per week for faecal samples and once weekly for blood and environmental for the course of the 3-month trial.

Large herd sizes are also associated with increased MAP infection, and since the removal of quotas in Ireland in 2015, herd sizes have continued to increase, thus threatening to impact the prevalence of JD. Although there are not yet studies to show that this is the case in Ireland, it has been well documented that an increased herd size has higher incidence of the disease [45]. A thorough examination of prevalence estimations have been examined between 2013 and 2014 [46], therefore, there is substantial data available to carry out a follow-up study post quota removal. In a comprehensive review by Whittington et al. (2019), a significant association between herd size and herd level prevalence was found, where, for every log increase in herd size, the odds of a country having a higher category of prevalence increased by 9.7% [47]. Examples of the effects of intensive farming on prevalence of MAP can be seen globally, with many studies examining factors which promote an increase in MAP positive herds. In a study carried out on 148 Canadian dairy farms, it was found that herds with > 200 cows were found to be more likely to be faecal culture, MAP positive and remained MAP positive for a number of years, than herds with < 51 cows [48]. It was also found that herds with > 200 cows had 3.54 times higher likelihood of a positive test in environmental samples than herds of < 50, again suggesting intensification has an impact on prevalence [49]. The level of intensity and its impact on herd and animal sero-prevalence of MAP was also examined by Liu et al. (2017) on farms in Northwest China and it was found that intensive farming (described here as herds with > 200 animals with no access to pasture) had a relatively higher risk of being infected with MAP than free ranging herds [45]. Multivariate logistic regression showed a significant association on the sero-positivity of goats from Spanish herds and intensive productions systems [50]. Ultimately, intensive production systems have a higher density of animals that are in close proximity to each other, thereby favouring horizontal transmission.

Control programmes have been developed and implemented in a number of countries worldwide in order to prevent the spread of the JD. Countries differ in the methods of control employed, with a number of programmes focusing on MAP-positive herds being tested regularly and the culling of infected animals. Other countries focus on MAP-negative herds and trying to keep those herds negative [42]. As there are no effective cures yet developed for JD, these control programmes are employed in order to try and eradicate the disease. Furthermore, the current vaccines for MAP compromises the diagnosis of bovine tuberculosis in cattle [5, 51]. JD vaccination programmes have been implemented in several countries including the USA, which previously used the commercially available vaccine Mycopar®, but have now been discontinued (2019). This is due to a number of short-comings with this particular vaccine including lesions at the site of inoculation and the interference with bovine TB (bTB) diagnostics. Additionally, the vaccine failed to completely prevent faecal shedding of the pathogen in a number of recent studies [52, 53]. Gudair® (Zoetis Inc.) is available in Australia and the UK for ovine Johne’s disease with Silirum® (Zoetis Inc.) available for bovine Johne’s disease (Australia only). Australia has an advantage in relation to vaccination programmes for JD in that bovine TB has already been successfully eradicated. Precautionary information available infers that the vaccine may have potential effects on false positive results to bTB testing and therefore would not be suitable in countries where *M. bovis* is still a problem. There have been 146 vaccine trials or studies conducted across a number of different countries which have been shown to positively impact the prevalence of disease in those countries [54]. Other countries, like Sweden for example prohibit by law the use of vaccination for JD [55]. The development of live attenuated vaccines, such as those examined by Shippy et al. [53], with an accompanying assay to differentiate between animals infected with *M. bovis* or MAP and vaccinated animals (DIVA), may offer a more robust and effective vaccination programme that may be applicable in countries that have not yet eradicated bTB. In countries where vaccination is not employed, the control of MAP infection is more difficult due to the fact that the pathogen can survive in ambient conditions for 8 months [56], for 19 months in water at 38 °C and in a desiccated state for up to 47 months [57]. This, combined with the slow progress of the disease, the slow growth of the bacteria and the sensitivity and specificity of current diagnostic methods, makes JD extremely difficult to eradicate. In a comprehensive review by Barkema et al. (2017), the many knowledge gaps that hamper the prevention and control of JD were highlighted [42]. The review identified many areas that need to be addressed in order to combat the disease including an assessment of the long term efficacy of control programmes, comparisons of MAP prevalence over time in the same regions, examination of the effect of mixed genotype infections and superinfections and the distribution of MAP genotypes, among many other areas of potential improvement.

### Diagnosis and detection of MAP

Effective control of animal diseases is dependent on rapid and accurate detection of pathogens using sensitive and specific diagnostic tests. There is a broad variety of methods available for the detection, identification and characterisation of pathogenic microorganisms present in a range of biological environments including blood, milk and faeces. These methods include microscopy, histopathological, isolation of pathogen from environmental samples, and immunological and molecular based diagnostics. These methods vary widely and each has their own individual benefits, in terms of cost, reproducibility sensitivity, specificity and time taken to produce results. Environmental testing is often used for herd level detection, where a slurry sample (urine, faeces and often, effluent) is used for MAP detection. This type of sampling may hamper the accuracy of the testing as a slurry mix provides a hostile environment for MAP survival. Hahn et al. (2017) examined the use of different diagnostic methods for the detection of MAP on boot swab and liquid manure samples [58]. Although the main aim of the study was to examine qPCR protocols for the detection of MAP, the group identified that liquid manure was negatively impacting on qPCR-based analysis, with boot swabs analysis being more representative of the infection status of the herd. The authors of this study also identified the dilution of MAP in the faecal slurry and/or the presence of effluents and urine that contribute to PCR inhibition as being factors in false negative results.

### Detection of MAP in cattle

#### Culture

Faecal culture is considered the “gold standard” in JD diagnostics [59]. It is relatively inexpensive and the specificity is considered to be almost 100%. However, this method relies on the growing of microbes, which is problematic as MAP is the slowest growing subspecies from the family Mycobacteriaceae, taking from 8 to 12 weeks for strains to grow. The Cornell double incubation decontamination method is often used, where a pre-incubation of the sample is carried out in brain-heart infusion medium to initiate the growth of bacteria and fungal spores. The second step involves the addition of antibiotics [60]. Furthermore, if animals are shedding low levels of MAP or are intermittently shedding the bacteria, sensitivity of culture can be low, ranging from 23 to 70% in infected, infectious and affected animals [61].

Liquid media of choice is typically Trypic Soy or Middlebrook supplemented with Oleic Albumin Dextrose Catalase (OADC) and mycobactin. Confirmation tests include the Ziel-Neelson staining method. This staining technique is used to stain acid-fast bacilli with an intact cell wall. Used as part of post mortem examinations, this test is cheap and fast, but cannot differentiate between mycobacterial species. The sensitivity of faecal culture is ~ 70% at the clinical stage of infection, but much less (23–29%) at the sub-clinical stage, where MAP is shed intermittently and in low numbers [62].

Isolation of MAP from the faeces of infected animals is still the most reliable method for detecting infected animals due to its high specificity. However, long incubation times and variations in sensitivity mean that this method also has its disadvantages. These variations may be a consequence of media type and methods of preparation i.e. commercial versus laboratory-made media, increasing inter-laboratory variability [63].

### Molecular epidemiology of *Mycobacterium avium* complex

**Fig. 1:**
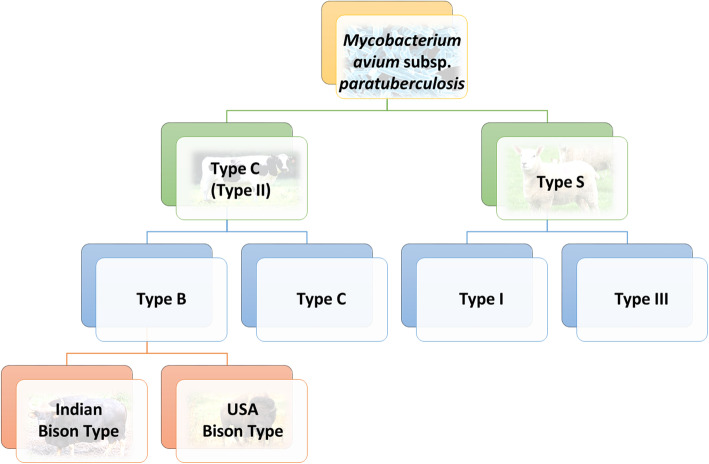
Nomenclature of *Mycobacterium avium subsp. paratuberculosis* (Adapted from [64]). There are two major groups of strains; Sheep-type, or Type S, and Cattle Type, or Type C, and an “intermediate” Type III, a sub-type of Type S. Type B is a subtype of Type C and is typically isolated from Bison

MAP is a subspecies of the *Mycobacterium avium complex* (MAC) along with a number of other opportunistic pathogens associated with both humans and animals (Fig. 1). Although genetically similar, these pathogens are distinct with respect to their hosts and pathogenic characteristics. The MAC consists of four subspecies, namely *Mycobacterium avium subsp. hominissuis* (MAH), *Mycobacterium avium subsp. avium* (MAA), *Mycobacterium avium subsp. salvaticum* (MAS) and *Mycobacterium avium subsp. paratuberculosis* (MAP). Research has shown that MAH is an environmental opportunistic pathogen found in humans and swine globally [65]. Soil and water are considered the natural reservoirs for this pathogen and, although isolated from a number of different animals, this does not confirm zoonotic potential [66]. MAH is considered the clinically most important of the MAC subspecies for humans [67], historically causing morbidity in acquired immunodeficiency syndrome (AIDS) patients. MAA causes a TB–like disease in birds, the main reservoir of the subspecies [68]. Avian TB is a chronic wasting disease, although in many cases is asymptomatic. Infection is thought to occur through fecal-oral transmission of contaminated faeces by susceptible animals. MAS is taxonomically close to MAA, causing a TB-like disease that occurs mainly wood pigeons but has also been reported in mammals [69, 70].

MAP itself can be further subdivided into Cattle Type and Sheep Type, each of which can be further divided as illustrated in Fig. 1. MAP is the only MAC subspecies that exclusively infects the gastrointestinal tract [71].

It is hypothesised that MAP originally evolved from MAH, which is the most genetically variable subspecies within MAC [72]. The proposed biphasic evolutionary model includes the idea of a proto-MAP, which evolved following the acquisition of 7 large sequences and the loss of 1 large sequence [65, 73]. The second phase, which involved the subdivision of the sheep and cattle type lineages, emerged independently following the lineage specific genomic deletion events. Alexander, Turenne and Behr (2009) examined the insertion and deletion events that shaped MAP as a subspecies, focusing on 25 large sequence polymorphisms, which are genomic regions present in some *Mycobacterium avium* (MA) strains and absent from others [73]. Sixteen of the regions examined were specific to MAP, with a number of the regions supporting the existence of lineage specific genovars, cattle type and sheep type. The LSPA20 and “deletion 2” regions were absent from strains of the sheep lineage only. Similarly, MAV-14, LSPA18, LSPA4-II, and a region of the GPL cluster were absent from all strains of the cattle lineage [73]. Another notable feature is that the MAP genome contains multiple copies (14–18) of a species-specific insertion element, IS*900*, that can be used for diagnostic purposes to, for example, detect the presence of the microorganism in faecal material following DNA extraction and PCR. This 1451 bp element is considered to be unique to MAP. The reference strain for MAP is K-10, a Type II (cattle type) strain, which a single circular chromosome consisting of 4,829,781 base pairs, encoding 4350 ORFs, and a G + C content of 69.3% [74]. The reference strain has 17 copies of the aforementioned IS*900* species specific insertion element. It is also worth noting that F57, a single copy insertion element, is also used for detection purposes [75, 76].

### Genetic diversity of MAP

Understanding the differences between strains of MAP is important in establishing how such difference might influence both the development and transmission of disease [77]. All microbial genomes are subject to plastic changing over time due to spontaneous mutations and in response to changing selective pressures within the microenvironments they inhabit. Understanding the genetic variability and, in turn, the molecular epidemiology of different strains of MAP can greatly improve our understanding of their influence on onset and severity of infection and also transmission patterns, enabling governments to employ more efficient control measures, tailored to each country.

In contrast to MAH, MAP has a relatively low genetic heterogeneity [64]. In total, 75% of the MAP genes have counterparts in TB, with 39 predicted proteins that are unique to MAP [3]. The genome possesses a high redundancy rate due to gene duplication, particularly for those involved in lipid metabolism and the redox process. As discussed earlier, there are two major groups of strains; Sheep-type, or Type S, and Cattle Type, or Type C, and an “intermediate” Type III, a sub-type of Type S [77], with several strain typing techniques available to categorise these isolates. Of these methods, it has been suggested that genome wide SNP detection provides considerable resolution between strains [78]. In contrast, although Mycobacterial Interspersed Repetitive Unit-Variable Number Tandem Repeat (MIRU-VNTR) can differentiate between Type C and Type S, it provides limited resolution between isolates within these lineages [78]. Regardless, molecular typing techniques have provided a valuable insight into the evolution, genetic diversity, interspecies transmission and inter and intra herd infection [59]. In one instance, de Kruijf et al. [79] examined a large collection of MAP isolates from the Republic of Ireland using MIRU-VNTR, and showed that among the isolates used, there were four distinct INMV group classifications (MIRU-VNTR patterns) reported from 53 herds. The most dominant groups found were INMV 1 and INMV 2, accounting for 98.2% of isolates analysed and highlighting a low genetic diversity among Irish isolates. This observation was consistent with another by Douarre et al. [80] also from Irish isolates. In a similar study carried out in German cattle herds, a high genetic diversity among MAP strains was apparent [81]. More recently, Bryant et al. (2016) explored the phylogenomic relationship between strains of MAP found in a range of different countries from a range of different hosts [78]. This study found that using whole genome sequencing (WGS) in conjunction with MIRU-VNTR led to better resolution. It was also found that sequencing of MAP isolates from patients with inflammatory bowel disease showed that these do not comprise a distinct strain type and cluster with Type C cattle isolates [78].

### Molecular methods

Molecular methods have been used for decades in microbiology research and have increasingly improved since their advent. They are rapid, sensitive and specific. As there are certain limitations associated with traditional microbiology techniques, a great deal of this can be overcome through the use of molecular methods. They are used to support and complement conventional methods. Current applications of molecular methods in human and veterinary medicine include the rapid use of non-culturable agents and antimicrobial drug susceptibility testing. These approaches offer a number of different protocols for the detection of disease caused by difficult to culture, slow-growing microbes. Results are obtained more rapidly, thus enabling prompt disease diagnosis. Amplification techniques such as polymerase chain reaction (PCR), real-time polymerase chain reaction (qPCR) or nucleic acid sequence-based amplification can be used for genotyping and quantification and can also be used in conjunction with culture techniques for confirmation of culture. Although these methods have significantly contributed to our understanding, they can be subject to error associated with cross contamination, ineffective DNA extraction protocols and the presence of inhibitors leading to false-positive/negative results. Cunha and Inácio (2014) have detailed a SWOT analysis of molecular diagnostic technologies [82].

### DNA extraction

DNA extraction methods affect the recovery of different microbial communities from environmental samples. Faeces represents one of the most complex matrices for microbial DNA isolation due to the presence of DNA from a number of other sources, namely food and the host itself, but also inhibitors which have consequences for PCR amplification and NGS preparation [83]. In order to apply PCR methods and the aforementioned sequencing approaches for microbiome analysis, the extraction of good quality DNA from faecal samples is pivotal for microbiome analysis [84]. A number of studies have compared a wide variety of commercial kits and protocols in order to determine the most sensitive and specific kit for carrying out molecular detection of MAP [84–87]. Extraction of genomic DNA from MAP is difficult due to its unique lipid rich cell wall, mentioned earlier in this review, making it difficult to lyse. This unique cell wall consists of a peptidoglycan layer, surrounded by a hydrophobic arabinogalactan-peptidoglycan-mycolic acid layer [71]. This is further surrounded by a second layer containing lipopentapeptide (L5P). A successful DNA extraction can be defined as one that results in high quantity and quality DNA. Therefore, the choice of suitable DNA extraction methods are pivotal in molecular research. Choice depends on a number of factors including time-efficiency, cost-effectiveness, the type of biological sample, collection and storage requirements [88, 89]. The use of mechanical disruption, such as bead-beating and/or sonication, can be used to increase the concentration of DNA achieved with sonication being shown to enrich mycobacterial DNA [90]. However, this shears background bacteria leaving it an unsuitable lysis method for microbiome analysis. The use of a pre-treatment enzymatic incubation to weaken the mycobacterial cell wall has been shown to increase in MAP DNA extracted. Lysis buffers, a heat lysis step and mechanical disruption being are necessary for the extraction of DNA from this difficult pathogen [76, 91]. Procedures for the removal of contaminants and DNA precipitation within extraction methods also play an important role in quality and quantity. Inefficient removal of contaminants, such as humic acids and complex polysaccharides, may result in PCR inhibition. DNA extraction efficiency has been compared using commercially available DNA extraction kits based on both magnetic separation and silica columns [87]. It was found that silica column based methods were superior to magnetic separation methods for both milk and faecal microbial DNA isolation. Efficacy was determined by qPCR. De Grossi et al. (2020) used a QIAamp DNA minikit following resuspension of faecal material in DEPC water [92]. This method did include mechanical disruption, which may be necessary for lysis of MAP cells. Bauman et al. (2016) [93] used the Tetracore MAP DNA extraction kits prior to decontamination protocols set out in a separate kit from the same company. The kit involves the use of mechanical disruption to lyse MAP cells. (Ramovic et al. 2019) [94] targeted the IS*900* sequence using the LSI VetMAX kit (LSI, Lissieu, France) and the spin column Qiagen DNA mini kit following double incubation. (Taniguchi et al. 2020) [95] used Johne-spin (FASMAC, Atsugi, Japan).

From our brief review of the recent literature it was found that commercial kits using mechanical disruption and silica column purification, due to their high binding affinity for DNA, are preferred for DNA isolation from complex matrices such as faeces and milk.

### qPCR

Quantitative PCR is now a well-established method for the detection, quantification and typing of microbial agents in clinical and veterinary diagnostics and food safety [96]. The main advantage of qPCR is that it can provide fast and high throughput detection of target DNA from complex biological matrices. This method allows for the quantification of targeted sequences where the number of amplification cycles required to generate a product correlates with the copy number of a target sequence.

The use of targeted regions of MAP such as the previously mentioned multi-copy IS*900* is commonly used in molecular MAP diagnostics, detecting MAP in culture, faeces, tissue, milk, milk powder and cheese. This is followed by f57, a single copy gene [63]. qPCR approaches have also been employed that target IS1311, *mbtA* gene, IS_MAP04 and IS_MAP02 [59, 97]. In a recent study carried out by Butot and colleagues, it was found that IS*900* targets were preferred in terms of sensitivity and presented with the lowest levels of variability between laboratories, for the detection of MAP in milk in comparison to f57 and phage based techniques. A number of commonly used primers can be found in Table 1. They state this is a consequence of the use of standardised reagents using commercial kits that are available for DNA extraction and qPCR assays. Digital PCR (dPCR), also known as third generation PCR, is said to be more precise and predict absolute numbers of microbes shed in faeces and is also resistant to inhibitors. Furthermore, a calibration curve is not necessary to provide a copy number, an issue that is often faced with qPCR. However, similar hurdles may be encountered during optimisation including choice of master mix, template properties and positioning of primers using the BioMark dPCR system. Nonetheless, dPCR may be an alternative to qPCR in the validation of tests and biomarkers. Such investigations have been carried out by Devonshire et al. (2015) with regards to *M. tuberculosis* [102]. More recent advances in rapid disease identification using PCR-like, lab-on-a-chip, portable devices are paving the way for cheaper and faster disease diagnosis. TriSilix, a disposable, silicon-based integrated Point-of-Need transductor, has been tested to quantitatively detect MAP using IS*900* primers, with the limit of detection equivalent to a single bacterium [103]. In that instance, DNA was extracted from pure cultures of MAP K-10 reference strain but further tests are needed with more complex matrices such as milk and faeces that contain inhibitors that may hamper results.

**Table 1:** Primer pairs, targets and conditions commonly used for MAP detection

Primer pair	Target	Size of product (bp)	PCR conditions	References
J5A (Forward 5′-ATGTGGTTGCTGTGTTGGATGG-3′) J5B (Reverse 5′-CCGCCGCAATCAACTCCAG-3′)	IS900	298	94 °C for 5 min; [94 °C for 30 s, 66 °C for 30 s, and 72 °C for 1 min] × 4 cycles; [94 °C for 30 s, 64 °C for 30 s, and 72 °C for 1 min] × 4 cycles; [94 °C for 30 s, 62 °C for 30 s, and 72 °C for 1 min] × 4 cycles, [94 °C for 30 s, 58 °C for 30 s, and 72 °C for 1 min] × 24 cycles, and a final extension at 72 °C for 7 min	[98]
IS900qPCRF(Forward 5′-GATGGCCGAAGGAGATTG-3′) IS900qPCRR(Reverse 5′- CACAACCACCTCCGTAACC-3′)	IS900	145	37 °C for 10 min, followed by initial denaturation at 95 °C for 15 min and 47 cycles of 95 °C for 5 s and 60 °C for 40 s	[99]
F57qPCRF (Forward 5′-GCCCATTTCATCGATACCC-3′) F57qPCRR (Reverse 5′-GTACCGAATGTTGTTGTCAC-3′)	F57	147	37 °C for 10 min, followed by initial denaturation at 95 °C for 15 min and 47 cycles of 95 °C for 5 s and 60 °C for 40 s	[99]
(Forward 5′ -CCGCTAATTGAGAGATGCGATTGG-3') (Reverse 5′ -AATCAACTCCAGCAGCGCGGCCTCG-3′)	IS900	229	1 cycle at 94 °C for 5 min and 20 cycles at 94 °C for 45 s, 58 °C for 1 min, and 72 °C for 2 min, followed by a final extension cycle at 72 °C for 7 min	[100]
f57_F (Forward 5′-TTG GAC GAT CCG AAT ATG T-3′) f57_R (Reverse 5′-AGT GGG AGG CGT ACC A-3′)	F57	254	1 cycle of pre-incubation: 95 °C for 10 min. Followed by 40 cycles of 95 °C for 10 s, 60 °C for 10 s and 72 °C for 20 s	[75, 97]
mbtA_F3 (Forward 5′–CTC CCG CAA CTC GGT CAC–3) MAP2179_R3 (Reverse 5′–CAC AGC CAG GTG TGA AAG–3′)	mbtA	307	1 cycle of pre-incubation: 95 °C for 10 min. Followed by 40 cycles of 95 °C for 10 s, 60 °C for 10 s and 72 °C for 20 s	[97]
P90 (Forward 5′-GAA GGG TGT TCG GGG CCG TCG GCC TTA GG- 3′) P91 (Reverse 5′-GGC GTT GAG GTC GAT CGC CCA CGT GAC-3')	IS900	394	4 min of initial denaturation at 95 °C, 37 cycles of 95 °C for 30 s, 59.5 °C for 30 s and 72 °C for 30 s, followed by a final elongation at 72 °C for 4 min	[31, 101]
AV1 (Forward 5'-ATGTGGTTGCTGTGTTGGATGG-3') AV2 (Reverse 5'-CCGCCGCAATCAACTCCAG-3')	IS900	N/A	95 °C for 10 min, followed by 40 cycles of 95 °C for 30 s, 58 °C for 1 min and 72 °C for 1 min	[28, 31, 101]

### Novel bacteriophage based methods

Bacteriophage, i.e., viruses that infect bacteria, can be used as tools for the detection of bacterial pathogens and offer a non-antibiotic method to improve animal health and food production. These viruses have co-evolved with their bacterial hosts to recognise and infect their target cells with extraordinary specificity that can be harnessed for rapid detection of MAP [104]. This is the due to the fact that the rapid, complete infection cycle of virulent phage can take just 1–2 h. Phage/phage lysis can be used in many ways to detect bacterial pathogens. This includes using intracellular components as markers to measure the lysis event and, in turn, the number of pathogen cells initially present. Adenosine triphosphate (ATP) is the intracellular marker most widely used for determination of bacterial numbers in a sample through cell lysis. Impedance or conductivity can also be used as a pseudomarker. Phage amplification assays can also be used, which involves the use of unmodified phage particles to generate and enumerate plaques within a bacterial lawn. The detection of *Mycobacterium tuberculosis* in human sputum samples, by using a lawn of fast-growing *Mycobacterium smegmatis* as an indicator, which constitutes the basic principle of the FastPlaqueTB and FastPlaque-Response (for detection of rifampicin-resistant *Mycobacteria*) tests, represents another interesting approach [105]. This assay has also been reported to be applicable to the detection of MAP upon optimisation [106]. The assay is coupled with plaque PCR testing for the presence of signature elements and allows to detection of viable cells [104]. The Actiphage® test developed by Swift et al [107], which uses the above principles [108], has been commercialised by PBD Biotech with a similar method recently developed by [101]. Phage based assays can also be coupled with peptide-mediated magnetic separation for MAP cell capture [109, 110]. However, the protocols are often complex and considerable training is required for accurate and reproducible results. Although phage based assays have the benefits of being rapid in comparison to more traditional methods, there are issues that may be encountered when attempting to isolate phage from a given environment. In order to generate phage, isolation of the host target bacteria is needed. This may be problematic when dealing with a slow-growing pathogen such as MAP. An avirulent MAP mutant or closely related fast growing, non-pathogenic host could also be considered.

### Host immunological response to MAP exposure; ELISA and Interferon gamma

An immunological response to MAP can be detected by measuring host antibody production for which a number of methods are available. However, they are only at their most sensitive in the late stage of infection when antibody production is highest. The enzyme linked immunosorbent assay (ELISA) is used to detect antibodies or infectious agents in a sample. In the case of JD, it examines the host response to MAP infection. However, the specificity and sensitivity of the test varies considerably, with sensitivity ranging from 15% in subclinical cases (due to low antibody production) to over 90% in clinical cases [111] ELISA of milk and serum samples is routinely used when screening herds for MAP [111]. Gamma interferon (IFN-γ) also provides an insight into cell mediated immunity against bovine tuberculosis and paratuberculosis. IFN-γ is a cytokine released by T-lymphocytes following the stimulation of the immune system by an antigen. Blood samples are taken from the animal and incubated in the presence of test antigens followed by a quantification of IFN-γ using ELISA. A difficulty associated with this type of testing is the presence of shared antigens with bovine tuberculosis and other members of the Mycobacteriaceae, causing animals which are not infected with paratuberculosis to cause false positive reactions. An investigation into this interference with test specificity was carried out by Kennedy et al. (2014). In the study, it was found that animals that were tested for bovine tuberculosis using the intradermal cervical comparative test interfered with JD ELISA diagnostics, resulting in a number of false positives. The sensitivity of serum ELISA is 40–87% in cattle with clinical signs, 24–94% in cattle with no clinical signs but shedding MAP and 7–22% in cattle with no clinical signs and no shedding of the organism. The sensitivity of this test is dependent on exposure to environmental mycobacteria, concurrent infection with *Mycobacterium bovis*, intradermal tuberculosis testing and MAP vaccination. It was also found that the administration of purified protein derivative as part of the bovine tuberculosis test, corresponded to an increase in the prevalence of ELISA positives for JD. The study recommended that a milk ELISA for JD should be avoided in the 43-day period following the administration of purified protein derivative, with serum sampling not recommended for an additional 28 days. Other studies have found that co-infection with the helminth *Fasciola hepatica,* which is known to exert an immunoregulatory response by down-regulating Th-1 responses in cattle, may also influence MAP infection. Naranjo-Lucena et al. (2020) examined the response of bovine immune cells to MAP using *F. hepatica* [112]*.* Although co-infection had a limited impact on the in vitro immune response of immune cells to MAP, co-stimulation using *F. hepatica* molecules appeared to have a measurable effect by reducing responsiveness of bovine monocyte derived macrophages ileocaecal lymph node leukocytes to MAP antigens or infection with MAP. The authors state that further work would need to be carried out in order to determine if co-infection would affect the progression of JD.

Ultimately, it is clear that the specificity and sensitivity of immunoassays varies and, in their current form, do not accurately detect MAP infected animals. Identification of MAP infection through changes in the microbial profile may lead to improved prognostics and diagnostics.

### The microbiome and MAP

Among the important roles of the microbiome is the regulation of the immune system [113–116]. Commensal microbes can regulate the immune response in eukaryotic hosts by inducing the inflammatory cascade via the nuclear factor- kappaB pathway (NF-κB) [117]. NF-κB is a key transcriptional factor controlling the expression of genes mediating inflammatory and anti-apoptotic responses. NF-κB is activated by toll-like receptors (TLRs), a class of membrane receptors that sense extracellular microbes through recognition of microbial products, trigger anti-pathogenic signalling cascades in intestinal epithelial cells and mucosal immune cells. TLR2, 4 and 9 are believed to play a critical role in the initiation of immune responses against mycobacteria [118]. TLRs are essential for the recruitment of immune cells and the initiation of adaptive immunity in other Mycobacterial diseases and can also be seen in mouse models [119].

Although not extensively researched, the microbiome of animals infected with MAP has been examined, although no consistent pattern has yet emerged. One such study of dairy calves showed an over-representation of the families Planococcaceae and Paraprevotellaceae and an underrepresentation of the genera *Faecalibacterium* and *Akkermansia* in the faecal microbiota of infected cattle [120]. The group also noted an enrichment of lysine and histidine metabolic pathways and an underrepresentation of glutathione metabolism and leucine and isoleucine degradation pathways within the ileal mucosa-associated microbiome of the MAP-infected cattle. Another study reported greater proportions of the genus *Psychrobacter* and reductions in the proportions of the genera *Oscillospira*, *Ruminococcus* and *Bifidobacterium* in cows infected with MAP [121], while in yet another [122], an altered fecal microbiota in cattle infected with MAP was also noted. More specifically, it was found that MAP positive animals had a higher abundance of *Arthrobacter* (from Actinobacteria) and Proteobacteria, *Alistipes, Paraprevotella* and *Bacteroides* were reduced in abundance compared to MAP negative animals. In the MAP negative animals *Firmicutes*, *Clostridium* and *Ruminococcus* showed low abundance whereas *Bacillus* and *Enterococcus,* that were highly abundant in the positive group, had decreased. Notably, in this study, MAP positive animals had a microbiota comprised of a 30% relative abundance of Actinobacteria while negative animals had just 0.1–0.2% abundance.

Ultimately, a more in depth analysis of the microbiomes of MAP positive relative to MAP negative animals, using shotgun sequencing to classify to species, or even strain, level may be pivotal in identifying microbiome-associated contributions to health and disease in the context of MAP. Importantly, the commensals within the gut microbiome can play an important role in the control of pathogens through the direct impact on the pathogens and/or the stimulation of host immunity. The impact of the microbiome on infection of animals with shiga toxin producing *E. coli* O157:H7 highlights this point [123], where groups of colonic bacteria were associated with pathogen shedding. Gamage et al. [124] reported that commensal bacteria influence *E. coli* O157:H7 persistence and shiga toxin production in the mouse intestine. Other examples include animals infected with *Campylobacter jejuni* [125], which shows similar changes to the microbiomes of animals infected with MAP, predominantly differences in Actinobacteria and Bacteriodetes (which are more abundant in infected animals relative to non-infected controls). It is not yet clear if the gut microbiota of ruminants has a role in preventing or contributing to MAP infection but, should such a role be established, there are multiple ways in which this knowledge could be applied. In particular, the investigation of potential interactions between the gut microbiota and MAP can be revealed through the use of carefully designed infection models.

Other –omics based approaches are also relevant. Metabolic profiling or metabolomics involves the identification and quantification of numerous low molecular weight compounds in biological fluid samples [126]. De Buck et al. [126] showed there was a significant increase over time in the metabolites allantoin, creatine, isobutyrate and tryptophan in MAP infected group, while acetone, isopropanol, glucose and myo-inositol decreased. This was the first study of its kind, with the aim of using individual metabolites or metabolic profiles as a novel early detection method for JD. The study showed that metabolic profiling detected changes associated with MAP infection quicker than diagnostics available at the time. Metabolomic profiling has also been used in humans suffering from IBD, ulcerative colitis and other gastrointestinal diseases with promising results [127–129].

The use of microRNAs (short, non-coding RNAs that regulate mRNA expression) also has the potential to be used as prognostic or diagnostic biomarkers for numerous human pathologies [130]. Fecal miRNAs can influence the composition of the microbiome and the microbiome can influence host physiology by affecting gene expression in host cells [131]. In a recent study by Shaughnessy et al. (2020), evidence of differential miRNA abundance in clinically affected versus healthy animals was observed [132].

The gut microbiota has also been shown to be influenced by host genetics. Many traits, such as carcass quality and milk yield, are associated with quantitative trait loci (QTL) or single nucleotide polymorphisms (SNPs). Several studies have identified loci associated with susceptibility to MAP infection. In a study by Kiser et al. (2017), three popular dairy breeds were examined for SNP associations with susceptibility to MAP tissue infection using allelic, additive, dominance and recessive genome wide association analysis models and 16 new quantitative trait loci were found in Jersey and Holstein populations [133]. In a more recent study by McGovern et al. (2019), it was found that genetic variation in Holstein Friesian in humoral response to MAP infection was present [134]. The idea of breeding for favourable traits such as higher milk yields and growth rates are not a foreign idea in livestock production. However, selecting for disease-resistant animals is somewhat novel and may prevent the contraction of MAP [134]. Being a member of the *Mycobacterium avium complex*, MAP is difficult to treat with antibiotics. A thick waxy cell wall means MAP cannot be penetrated by cell wall targeting antibiotics. Therapeutic agents such as penicillins, vancomycins and cephalosporins are rendered inadequate as they target peptidoglycan biosynthesis. In fact, the use of such therapeutic agents may confer antibiotic resistance and alter the gut microbiota, potentially further contributing to the disease and susceptibility to other diseases. Antimicrobial combinations are used to treat and delay onset of the disease, consisting of macrolide protein synthesis inhibitors such as clarithromycin and azithromycin combined with ethambutol. These agents affect cell metabolism along with rifamycin which inhibits RNA synthesis [135]. As JD is a slow onset disease with many stages before the animal becomes clinical, it can often be mistaken for other infections, and susceptibility to other infections, leading to the treatment with ineffective antibiotics.

The gut microbiome is thought to have a considerable impact on human and animal health [117, 136–139], and research in this area has led to the identification of novel microbiome biomarkers for disease [120, 140, 141]. There has also been a renewed focus on manipulation of the microbiome through diet or the provision of microbes as probiotics or biotherapeutics for disease control. Defining a core healthy microbiome can prove difficult as many different factors can play a role in shaping the microbiome. Diet, for example, plays a pivotal role in shaping the gut microbiota [142–144], with many different feeding systems (high-grain diets versus grass-based systems) applied worldwide. Animal husbandry practices (indoor versus outdoor systems) and environmental stresses [145] may also be important. All of the above parameters are applicable in both healthy and diseased animals. It is also noteworthy that the metabolic functions of the microbiome also vary across healthy and diseases states, therefore potentially providing additional biomarkers.

The point is frequently made that hosts have co-evolved with the gut microbiome in order to achieve stability, thereby creating “superorganisms”, in which the microbiome performs many immune, metabolic and other functions [146]. The composition and function of the livestock gut microbiome has been extensively investigated in recent years, with a large focus on the bovine/ovine rumen microbiome from the perspective of methane mitigation strategies, improved feed utilisation and overall health and production performance. The rumen is the forestomach of ruminants and is a large anaerobic, methanogenic fermentation chamber responsible for providing nutrients to the host animal, contributing to end-product yield and quality. Dietary changes can result in a shift in microbiome composition and diversity, affecting the levels of volatile fatty acids (VFAs) produced by microbes. VFAs are the product of a series of fermentation reactions and are the primary source of energy for ruminants. VFAs such as acetate, propionate and butyrate are produced by bacteria. Their ratios influence feed efficiency, animal health and enteric methane emissions and have been the focus of extensive examination from the perspective of the rumen microbiome [147–151]. Butyrate, the main energy source for epithelial cells in the gut, in particular, plays an important role in host physiology and gut health, interacting with the immune system and providing anti-inflammatory effects in humans [152, 153]. Indeed, a reduced abundance of butyrate producing species such as *Faecalibacterium prausnitzii*, *Ruminococcus spp.* and *Eubacterium spp.* has been observed in patients with CD [21], and have the potential to serve as non-invasive faecal biomarkers. VFAs may also have potential with respect to MAP detection and JD, however, more research is needed in this area.

**Fig. 2:**
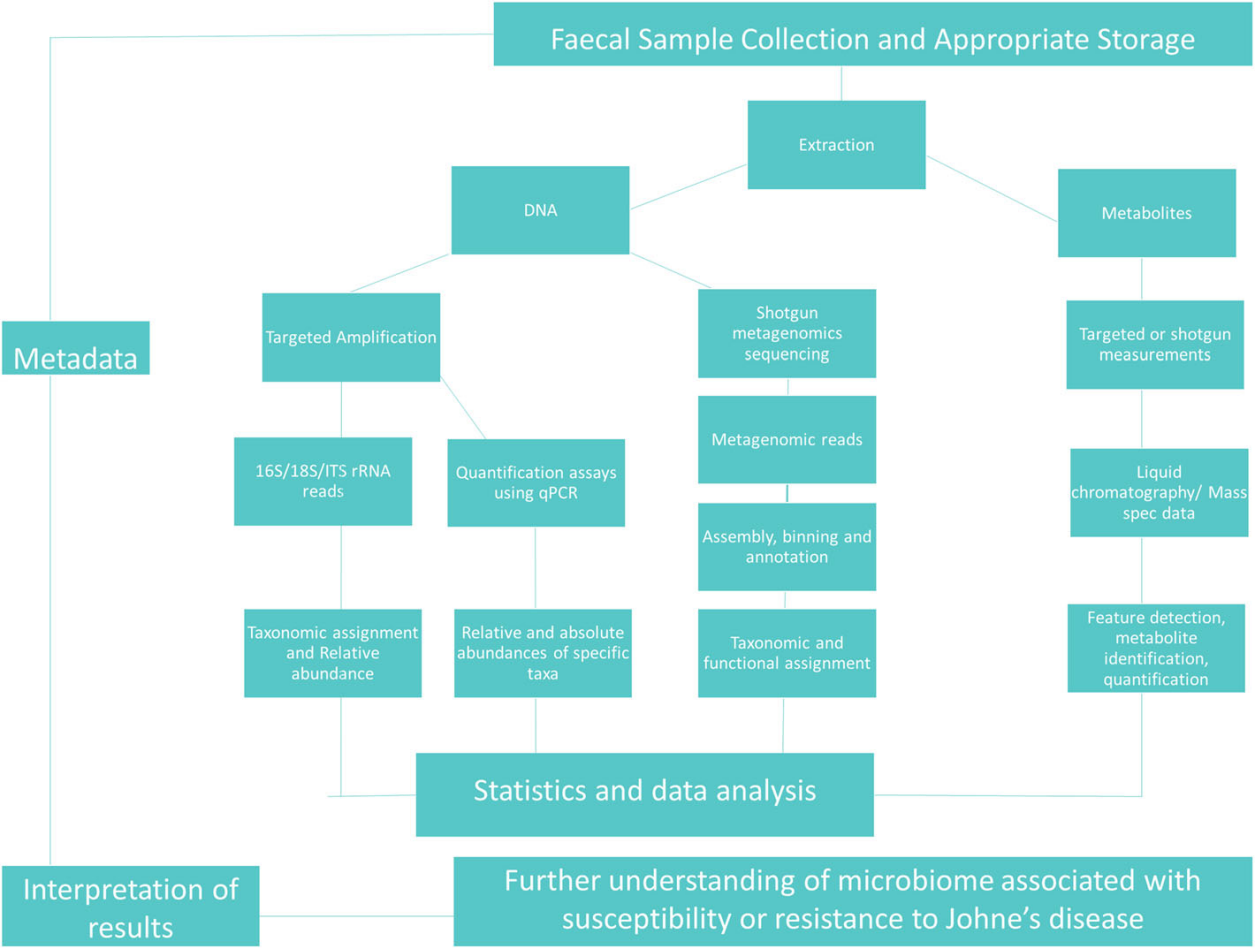
Microbiome project workflow for determining the microbiome associated with JD susceptibility or resistance (Adapted from Matthews et al. [160])

The post-ruminal digestive tract is also of considerable importance, particularly with respect to gastrointestinal diseases, but research in this area is relatively less advanced. The microbial composition of the post-ruminal digestive tract depends largely on pH, gut motility, redox potential and host secretions within the different compartments [154]. Significant differences have been noted between the microbiome of the small intestine (duodenum, jejunum and ileum) and the large intestine (cecum, colon and rectum) [155] and data also indicates that luminal and mucosa associated communities also differ among regions and may influence shedding patterns of pathogens such as *E. coli* [156, 157]. With this in mind, sampling methods may also produce variable results. This may include differences in collecting samples directly from the rectum, making contact with the mucosa or a free-fall sample which will be exposed to the external environment. Sampling at necropsy, which would allow access to multiple sites along the GIT, would be representative of particular site, allowing accurate comparisons. The post-ruminal digestive tract is composed mainly of bacteria, however methanogenic archaea have also been observed [155, 158, 159]. Figure 2 illustrates a typical microbiome project workflow, from sample collection through to data analysis and interpretation. Employing high throughput DNA sequencing techniques enables the detection of specific microbes and functional genes associated with healthy or diseased states, including the use of microbiome signatures as biomarkers, at different locations in the gastrointestinal tracts of ruminants.

### Potential future directions

Changes in the gut microbiome can be associated with disturbed gut barrier function and increased gut permeability [153]. Therefore, inducing the colonisation of protective bacteria may have a role in protecting against MAP-induced gut inflammation. Direct fed microbials, i.e. probiotics, have the potential to be used as a novel treatment for JD. In a study by Karunasena et al., (2013), the use of *Lactobacillus animalis* NP-51 was examined for its effect on animal health in an in vivo murine model infected with MAP. It was found that the probiotic reduced the production of cytokines associated with the increased stimulation of macrophage [161]. This may be a direct effect of the probiotic or be mediated through the microbiota. Indeed, it was notable that a positive correlation was identified between the gut microbiota composition and the aforementioned host immune responses. More specifically, it was found that, in untreated mice, the phylum Bacterioidetes correlated with an interferon gamma response and Proteobacteria correlated with levels of interleukin 6. Although intriguing, much more research is needed in this area. The identification and growth of novel biotherapeutic strains that inversely correlate with MAP levels or disease status may be used for prevention or treatment. Lactic acid bacteria, with species belonging to several genera including *Lactobacillus, Lactococcus, Enterococcus, Pediococcus, Streptococcus*, and *Leuconostoc* offer promise to finding the most suitable and beneficial microbes for livestock [162]. Isolating and growing these microbes may prove difficult due to their high dependence on a culmination of specific environmental niches, which may only be found in the gut. Direct inoculation of an entire faecal microbiome, through faecal microbiota transplant, may offer another means.

Feacal microbiota transplantation (FMT) is another, more extreme, option. The method is effective in treating disease as it increases microbial diversity, increases the prevalence of beneficial microbes and aids in restoring a normal microbiome, which may be able to modulate immune response. The beneficial microbes that are re-introduced may outcompete pathogens through competitive exclusion [163]. The procedure has been used to treat *Clostridium difficile* infections in humans, when all other methods have been exhausted. The method has proved highly successful in clearing infection. FMTs are received from healthy donors which are then used to treat *C. difficile* infection. Brown et al. [164] investigated the microbial community dynamics and metabolic changes associated with successful FMT, finding the microbiome of *C. difficile* patients became more similar to the microbiome of the healthy donors. Similarly, transfaunation is a method used in ruminants to re-establish or alter the microbiome in the rumen, where rumen contents is used rather than faeces. Rumen contents is taken from one healthy animal and transferred into an unhealthy animal. Historically, it has been used to treat digestive issues, but more recently has been used to examine feed efficiency [165]. Previously, it was found that the rumen microbiome was host specific, with the rumen returning to its original state following transfaunation [166]. Zhou et al. [165] found that certain bacterial phylotypes, namely *Lactobacillus*, *Coriobacteriaceae* and *Coprococcus* may have higher manipulation potential by means of content transfaunation, however, overall the microbiome remained host specific. Ribeiro et al. [167], found that two separate inoculations of rumen contents from bison to cattle was successful in altering the microbiome across time. In a comprehensive review by Niederwerder, (2018) the use of FMT in veterinary medicine was examined and broken into three potential applications; therapeutic use, prophylactic use and for stimulating pathogen-specific immunity and was examined for both ruminants and monogastrics. The area is currently in its infancy in veterinary medicine but may be an emerging prophylactic tool for use in the fight against JD.

As previously described by Barkema et al. [42], the effect of MAP genotype on disease progression, shedding and immune responses is not well characterised. This may open promising avenues for prognosis following further investigation. Analysis of volatile organic compounds produced during culture may assist in identifying growth and also strain identification [168]. This may include the use of methods more traditionally used for methane detection in ruminants during greenhouse gas emission studies should specific gaseous emissions associated with MAP infection be identified.

Emerging technologies such as biosensors, mobile phone applications and satellite data may provide novel methods for disease detection and control in the coming years. On-farm disease detection may be applicable using portable sequencing devices such as Oxford Nanopore’s MinION. McCabe et al. [169] used the technology for on farm detection of viral pathogens associated with bovine respiratory diseases. The authors describe the device as a Mk1B is a pocket-sized (105 mm × 23 mm × 3384 mm, 87 g) field-deployable sequencing device that is based on nanopore sequencing, mentioned earlier in this review. Rapid extraction and sequencing kits would need to be optimised for the detection of MAP using these kits. Mobile apps can be used to connect farmers, veterinary practitioners and scientists. One such app, EMPRES Global Animal Disease Information system has been designed to assist veterinary services by facilitating regional and global disease information. The tool has contributed to a better understanding of influenza epidemiology and ecology in livestock [170]. Developing a similar tool for JD control, where parameters including, but not limited to, host genetic data, mode of birth of animal, microbiome data, MAP isolate data, on farm soil chemistry, composition and soil microbiome composition and sward type may contribute to a better understanding of disease susceptibility, resistance and transmission. This would require a multi-disciplinary approach in order to create such a database. The Big Data approach to animal health and welfare, using computer modelling and statistical techniques, will improve welfare, production and sustainability, contributing to a planetary health strategy to reduce the threat of infectious disease, minimise environmental footprint and promote nutrition [171].

## Conclusions

MAP was first reported to cause JD in ruminants in the late 1800s. At that point it was described as intestinal tuberculosis and known as pseudotuberculous enteritis. Since then, the understanding of the disease has grown immensely, with the bacteria causing the disease having been the subject of detailed genomic analysis. In parallel, our appreciation of the role of the gut microbiome in human and animal health has advanced considerably. Understanding its role in complex gastrointestinal diseases, such as JD, could lead to preventative measures and the development of novel therapeutic agents such as probiotics. Dietary interventions may also play an important role in minimising the detrimental effects on the animal. While the microbiome of MAP infected animals is understudied, it has the potential of contributing enormously to the understanding of this complex disease, as has recently been seen with human microbiome studies on human disease. Using the knowledge achieved from such studies may provide a foundation to work off, building on that knowledge to advance the understanding of complex gut disorders.

## Data Availability

N/A

## Abbreviations

ATP: Adenosine triphosphate
CD: Crohn’s Disease
cfu: colony-forming unit
DNA: Deoxyribonucleic acid
ELISA: Enzyme linked immunosorbent assay
FMT: Fecal microbiome transplant
FODMAPS: Fermentable oligo-, di-, mono-saccharides and polyols
GIT: Gastrointestinal tract
IBS: Irritable bowel syndrome
IFN-γ: Gamma interferon
ITS: Internal transcriber spacer
JD: Johne’s Disease
MAA: *Mycobacterium avium subsp. avium*
MAC: *Mycobacterium avium complex*
MAH: *Mycobacterium avium subsp. hominissuis*
MAP: *Mycobacterium avium subsp. paratuberculosis*
MAS: *Mycobacterium avium subsp. salvaticum*
MIRU: VNTR - Mycobacterial Interspersed Repetitive Unit-Variable Number Tandem Repeat
NF kB: Nuclear factor- kappaB pathway
NGS: Next generation sequencing
OADC: Oleic albumin dextrose catalase
ORF: Open reading frame
PCR: Polymerase chain reaction
qPCR: real-time PCR
QTL: Quantitative trait loci
SNPs: Single nucleotide polymorphisms
TLRs: Toll-like receptors
VFAs: Volatile fatty acids
WGS: Whole genome sequencing
